# Controlled release strategy of paclitaxel by conjugating to matrix metalloproteinases-2 sensitive peptide

**DOI:** 10.18632/oncotarget.10735

**Published:** 2016-07-20

**Authors:** Changjiang Huang, Xiulin Yi, Dexin Kong, Ligong Chen, Gong Min

**Affiliations:** ^1^ School of Chemical Engineering and Technology, Tianjin University, Tianjin, China; ^2^ Tianjin Institute of Pharmaceutical Research, Tianjin, China; ^3^ School of Pharmacy, Tianjin Medical University, Tianjin, China; ^4^ Department of Oncology, University of Oxford, Oxford, UK

**Keywords:** matrix metalloproteinase, tumor targeting peptide, drug conjugate, paclitaxel, tumor metastasis

## Abstract

Peptide drug conjugates offer a novel strategy to achieve controlled drug release. This approach avoids the clinical obstacles of non-specific toxicity and overall drug resistance of conventional cytotoxic agents, such as paclitaxel. MMP2 plays important functions in tumour proliferation and metastasis. Herein, we conjugated the paclitaxel with a hexapeptide which is specific recognized by MMP2 protein. The conjugate is dissociated upon the MMP2 specific proteolysis at COOH terminal of hexapeptide, PVGLIG.

The results clearly indicated that the PVGLIG-paclitaxel conjugate significantly enhanced the tumor specificity against HT-1080 and U87-MG tumour cells. Our finding suggested that the hexapeptide PVGLIG is capable to act as a controlled and sustained drug carrier of paclitaxel for the treatment against tumour proliferation and metastasis with high MMP2 expression.

## INTRODUCTION

Matrix metalloproteinases (MMPs) are well known, as they play important functions in tumour proliferation and metastasis [1–3]. In the past decade, many studies have demonstrated that over-expression of MMP2 and MMP9 plays a vital role in metastatic tumour cells, promoting tumour growth and angiogenesis in the tumour, thereby providing nutrition to the tumour through the newly generated vessels [4–7]. In animals, MMP2 inhibitors prevented tumor dissemination and the formation of metastases by anti-angiogenic properties [8–12].

The specific protease activity of MMPs has made it an attractive approach for controlling drug release. The MMPs associated peptide drug conjugate (PDC) is designed for enzymatically metabolized prodrugs, in which therapeutic drugs are covalently bound to MMP substrate peptides [13–15]. The prodrug is then dissociated with the peptides by proteolysis of MMPs. Recently, several MMP associated PDCs have been developed in order to improve the tumor specificity of small chemical agents, including paclitaxel, doxorubicin, methotrexate and so on [16, 17]. In contrast to the original therapeutic approach of using small molecular inhibitors of MMPs, these novel drug-targeting strategies resulted in an improved therapeutic index and less toxicity [18–20].

In this study, a novel MMP2 specific hexapeptide was screened out using phage display technology, and then paclitaxel was conjugated. Initially, the antitumor activities of conjugate were investigated *in vitro* and *in vivo*. Data indicated that the conjugate possessed specificity for various tumour cell lines and tumour-bearing mice. In addition, the release of paclitaxel from conjugate was analysed using LC-MS which is crucial to overcome the main obstacle in the development of MMP2 associated drug candidate.

## RESULTS

**Figure 1:**
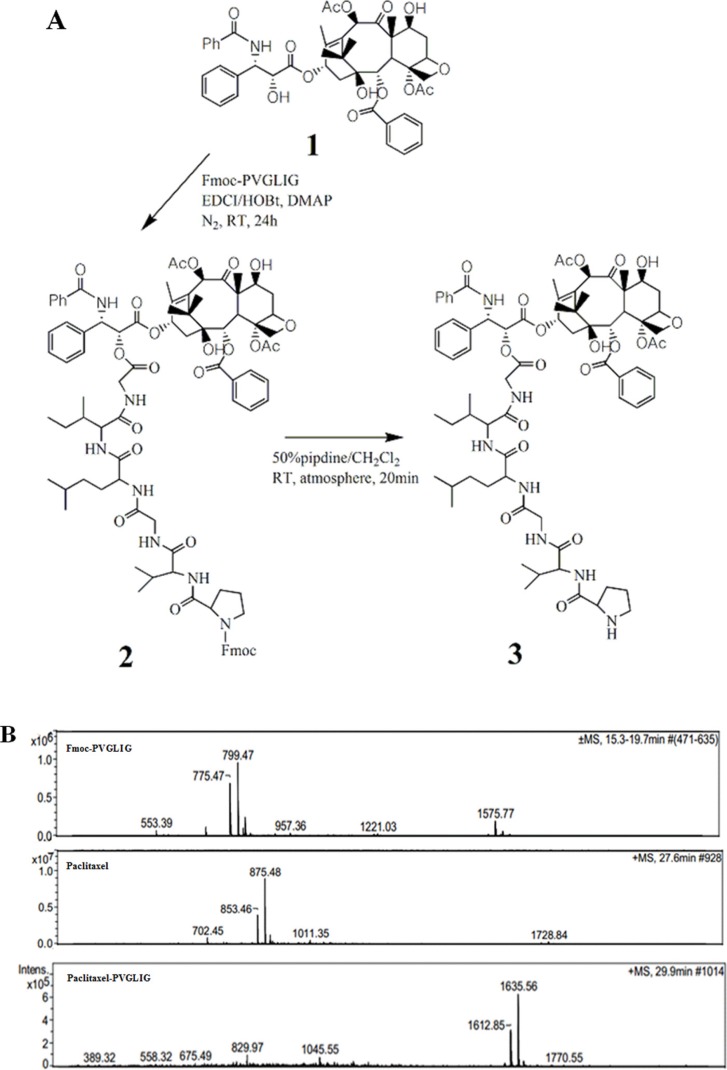
Synthesis of conjugate (**A**) Synthetic scheme of conjugate (**B**) Mass spectra observed in synthesis procedure Legend: Peptide was prepared containing an N-terminal Fmoc protection group in order to avoid the by-product. The Fmoc group is removed finally using traditional protocol. The samples were analyzed for ascertain the intermediate and final product (1428.34) using mass spectrometry. Conditions: Fmoc-PVGLIG was synthesized using solid phase peptide synthesis protocol and the purified product was then reacted with free paclitaxel. The final product was purified by RP-HPLC with XBridge C18 silica column and ascertained by HPLC-MS.

A novel hexapeptide, PVGLIG, was screened out since its high binding affinity to MMP2 protein. The peptide was then conjugated with paclitaxel for achieving improvement on tumor cells selectivity. In this study, paclitaxel was chemically linked to the COOH-terminus in peptide (Figure 1). Mass spectrophotometry was performed in order to monitor the reaction and to identify the final product (Figure 1).

**Figure 2:**
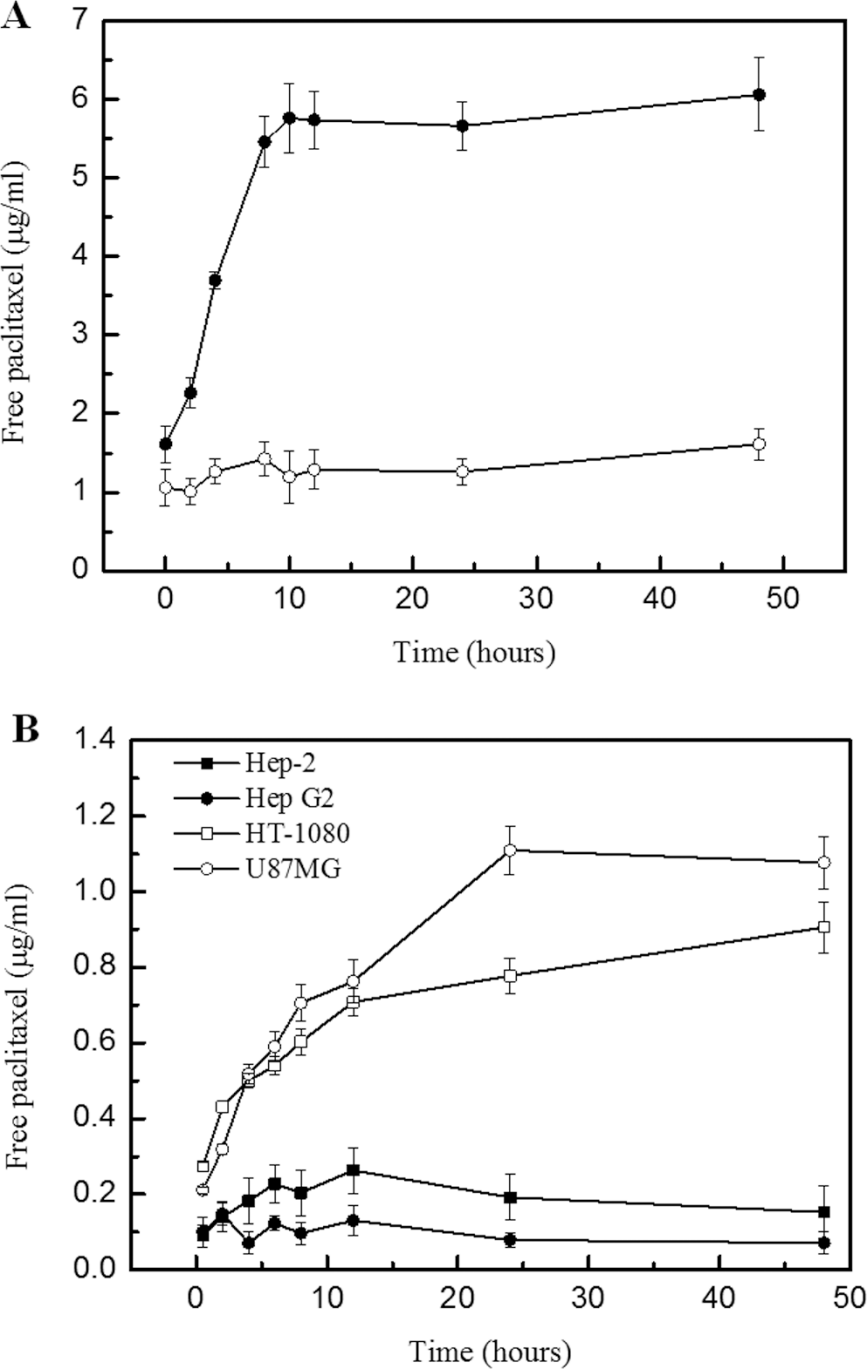
Paclitaxel dissociated from conjugate in MMP2 solution and various cell lines (**A**) Concentration of released paclitaxel from conjugate upon the MMP2 proteolysis (**B**) Concentration of released paclitaxel from conjugate incubating within various cell lines. Legend: Result indicated that the presence of MMP2 enzyme induced the release of paclitaxel from conjugate significantly. The conjugate initiated the drug release at 2 h after incubation with MMP2 and the level of dispatched paclitaxel peaked to plateau at 12 h (Panel A). In addition, the conjugate was incubated with various cell lines and the released paclitaxel was monitored by HPLC-MS. Results indicated that the conjugate showed different drug dissociation characterization in various tumor cell lines. The MMP2 over expression tumor cells, HT-1080 and U87MG, induced the release of paclitaxel remarkably, compared with those from Hep-2 and Hep G2 cells (Panel B). The data confirmed that conjugate exhibited the presumed MMP2 sensitive activity. Condition: Conjugate containing 100 μg paclitaxel were incubated with MMP2 (5 μg) in PBS buffer, Ph 7.0 containing 100μM ZnSO_4_ at 37°C for 48 h-experimental period. Various cells were seed in 96-well plate, and conjugate containing 10 μg paclitaxel was titrated into each well for incubation at various experimental periods. HPLC-MS was then employed to monitor the released free paclitaxel.

A clear understanding about the dissociation of conjugate conjugate *in vitro* is necessary to ascertain the appropriate dose of the conjugate in further animal experiments. Initially, the conjugate was incubated with MMP2 protein and the free paclitaxel was monitored. Result indicated that the free paclitaxel was started to release from conjugate 2 h after incubation with MMP2 protein. The concentration of free paclitaxel peaked to a plateau at 4–12 h (Figure 2A). The dissociation pattern of conjugate incubated with HT-1080 and U87MG cells exhibited similar feature with that of incubation with MMP2 protein, (Figure 2B). However, there is almost none of free paclitaxel dissociated from conjugate with in the 48 h incubation with Hep-2 and Hep G2 cell lines (Figure 2B). The specificity of conjugate in HT-1080 and U87MG tumor cells was derived from various expression levels of MMP2 in cells.

In addition, the cellular MTT and scratch assays were performed for evaluating the inhibition activity of conjugate on tumor metastasis. From MTT assay, shown in Table 1, result indicated that the conjugate exhibited improved cell viability against HT-1080 and U87MG tumor cells compared to paclitaxel control. In coincidence, there were no remarkable differences observed from Hep-2 and Hep G2 cell lines treated with conjugate or paclitaxel alone.

**Table 1:** MTT assay of conjugate in tumor cells, in comparison with that of free paclitaxel

	HT-1080		U87MG	
Time	Paclitaxel	Conjugate	Paclitaxel	Conjugate
24 h	19.71 ±2.61	42.73 ± 2.69	21.18 ± 2.94	38.15 ± 1.82
48 h	28.48 ± 3.11	53.16 ± 1.85	36.85 ± 4.27	62.16 ± 3.04
72 h	37.89 ± 2.19	84.24 ± 2.43	50.17 ± 2.64	87.83 ± 4.26

	Hep-2		Hep G2	
Time	Paclitaxel	Conjugate	Paclitaxel	Conjugate
24 h	21.05 ± 2.45	22.74 ± 3.62	20.99 ± 1.93	17.54 ± 1.03
48 h	30.65 ± 3.45	27.85 ± 3.75	45.35 ± 3.04	20.11 ± 2.65
72 h	46.76 ± 3.84	30.15 ± 3.85	51.34 ± 3.96	34.74 ± 2.94

Condition: The cells (1 × 10^5^ cells/100 μl/well) were seeded in 96-well plates at 37°C and 5% CO_2_. An aqueous solution of free paclitaxel and conjugate containing 10 μg paclitaxel were added in the culture medium following MTT assay protocol. Legend: In MTT assay, the anti-proliferative activity of conjugate was confirmed to be MMP2 specific. In HT-1080 and U87MG cell lines, where MMP2 was highly expressed, the treatment of conjugate resulted in significant loss of cell viability, compared to Hep-2 or Hep-G2 cells with less MMP2 expression.

**Figure 3:**
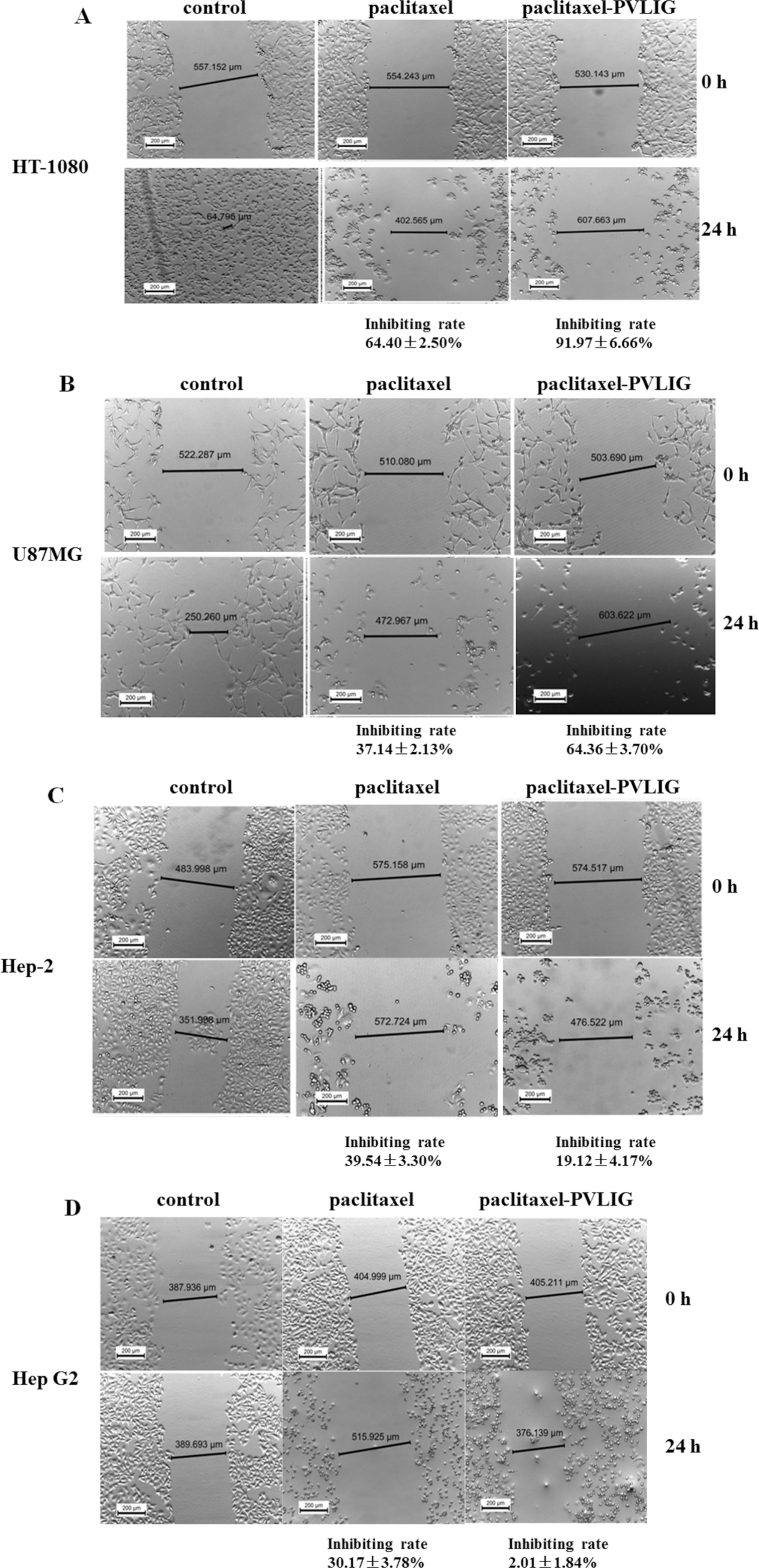
Conjugate exhibited remarkable anti-proliferation activity on specific tumor cells (**A**) Scratch assay of free paclitaxel and conjugate on HT-1080 cells (**B**) Scratch assay of free paclitaxel and conjugate on U87MG cells (**C**) Scratch assay of free paclitaxel and conjugate on Hep-2 cells (**D**) Scratch assay of free paclitaxel and conjugate on Hep G2 cells. Legend: Compared with the original migration inhibition effect of free paclitaxel in HT-1080 and U87MG cells, the conjugates presented a 1.4-fold and 1.7-fold increase on anti-migration activity, respectively. However, the conjugate failed to show anti-migration activity on Hep-2 and and Hep G2 cells those non-expressing MMP2. It was assumed that the inhibitory activity of conjugate on the cellular migrating phenotype is due to the specific release of paclitaxel upon the presence of MMP2 enzyme. Condition: The HT-1080, U87MG, Hep G2 and Hep-2 cell lines were plated in 6-well plates and cultured overnight respectively. The cell monolayer was scraped in a straight line with a sterile p200 pipette tip, the debris were removed by gently flushed with PBS twice. After rinsing, 2.5 mL cell supernatant (supplemented with 5% FBS) containing either paclitaxel or conjugate (final concentration of each treatment drug at 5 mg/ml) was covered on the scraped cells and then incubated cells at 37°C, 5% CO_2_ for 24 hours. The distances of cellular edges were measured by using LEICA ICC50 HD microscopy (LEICA Microsystems Ltd, Switzerland).

As known, MMP2 plays a critical role in tumour progression, tumour angiogenesis and metastasis; thus a cellular scratch assay was performed for investigating the metastasis inhibition of conjugate. In Figure 3, paclitaxel exhibited anti-proliferative activity against almost all tumour cell lines without any selectivity. However, the metastasis inhibiting rate in HT-1080 and U87MG cells followed treatment of conjugate was increased significantly than that of paclitaxel (Panel A and B in Figure 3).

**Figure 4:**
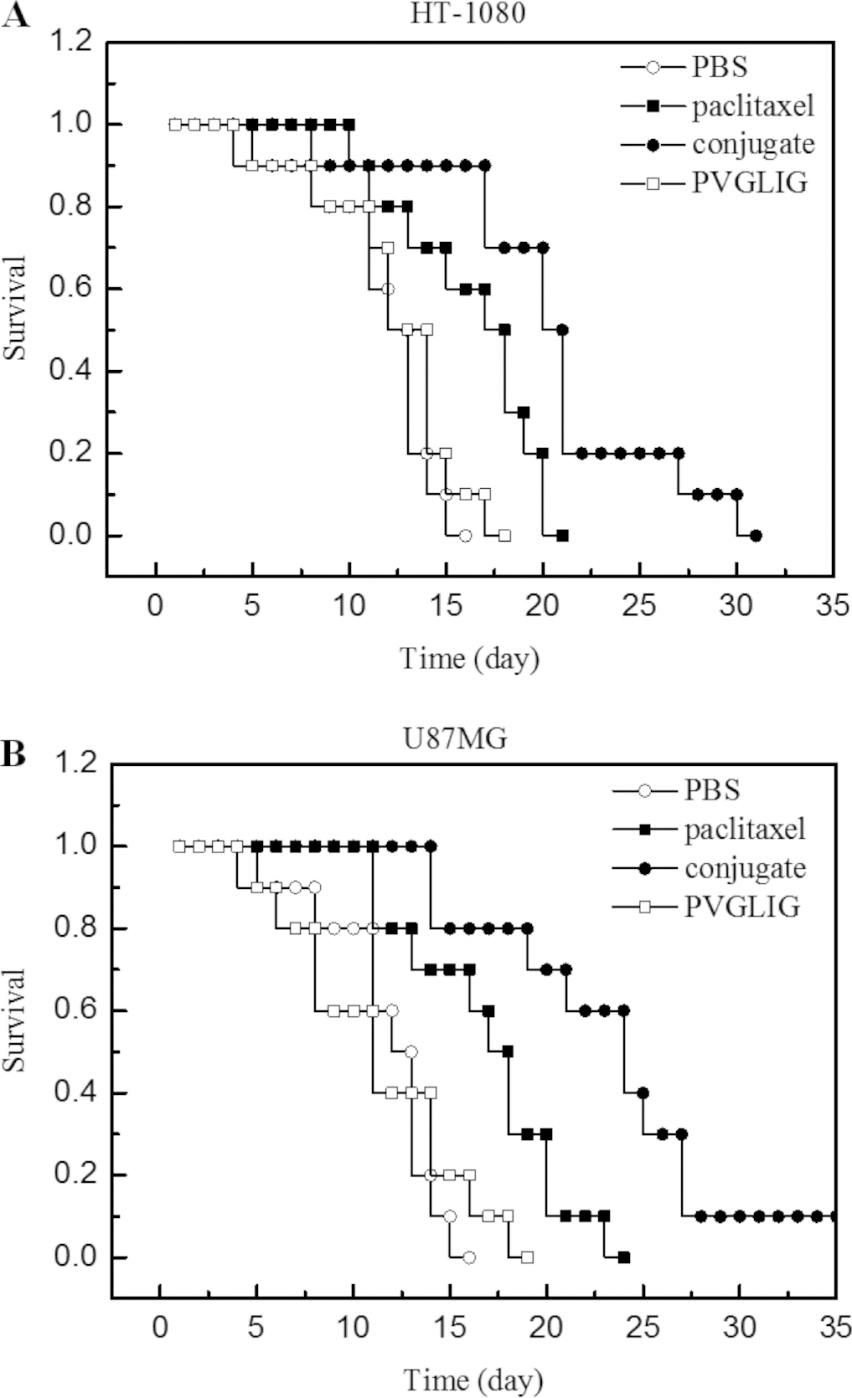
Anti-tumor efficacy of conjugate on human tumor bearing mice (**A**) Effect of conjugate on human xenograft tumor model mice bearing HT-1080 cells (**B**) Effect of conjugate on human xenograft tumor model mice bearing U87MG cells. Legend: Results indicated that the conjugate (●) exhibited an improved survival time compared to free paclitaxel (■) in either HT-1080 (Panel A) or U87MG (Panel B) bearing mice, *p* < 0.05 (*n* = 10). Condition: Xenograft mice bearing HT-1080 and U87MG were treated with the conjugate and free paclitaxel, respectively. Survival time was recorded in days after tumour injection. All data obtained for repeated experiments were pooled and utilized for statistical analysis.

To evaluate the anti-tumour effect of conjugate and free paclitaxel, the mice bearing with tumours derived from either HT-1080 or U87MG cells were treated with conjugate (15 mg paclitaxel/kg body weight/5 day) and free paclitaxel (15 mg/kg body weight/5 day), respectively. The antitumor activities were determined by monitoring the animal survival rate during 30 days treatment. The results in Figure 4 revealed that the administration of conjugate significantly increased the survival rate of experimental mice.

In summary, an MMP2 associated drug release system was developed based on a novel MMP2 specific peptide substrate in this study. The hexapeptide, PVGLIG, was conjugated with paclitaxel at COOH-terminal of peptide. This conjugate is capable to release paclitaxel for its conventional cytotoxic activity upon the presence of MMP2. This novel drug release system was considered to increase the therapeutic index of paclitaxel due to the enhanced specific targeting activity.

## MATERIALS AND METHODS

### Materials

Fmoc-amino acids and resins in this study were purchased from GL Biochem Ltd. (Shanghai; HPLC-purified; purity > 99%, identified by mass spectra). Paclitaxel was obtained from Demochem Co (Shanghai, China). All other chemicals were obtained from Sigma-Aldrich unless otherwise noted. The human recombinant MMP2 was purchased from Biomol International, Inc (Plymouth, PA).

### Cell culture

The HT-1080, Hep G2 and MCF-7 cells were cultured in DMEM (Gibco by Invitrogen, California, USA) supplemented with 10% fetal bovine serum (Gibco by Invitrogen, California, USA). The U87MG cells were cultured in MEM (Gibco by Invitrogen, Carlsbad, California, USA) and RPMI-1640 (Gibco by Invitrogen, Carlsbad, California, USA) supplemented with 10% fetal bovine serum (Gibco by Invitrogen, California, USA). All cell lines were cultured at 37°C in an atmosphere of 5% CO_2_.

### Synthesis of peptide (Fmoc-PVGLIG) and conjugate

The peptide was synthesized by solid-phase synthesis using a CEM Liberty 1 peptide synthesizer. The synthesized peptide was purified by reverse (XBridge, 5 μm particle size, 10 × 100 mm; Waters, USA). The purified sample was evaluated by mass spectrometry. In brief, 12.5 mg EDCI (66.1 μmol) and 8.8 mg HOBt (66.1 μmol) were titrated into a paclitaxel solution (56.5 mg, 66.1 μmol) followed by the addition of 2 mg DMAP after 15 minutes of stirring under nitrogen atmosphere at ambient temperature. After another 5 minutes of stirring, the peptide (77.5 mg, 99.7 μmol) was added and then stirred for 24 hours. The reaction mixture was dried under reduced pressure following piperidine treatment for the removal of Fmoc group. RP-HPLC using an XBridge C18 silica column (5 μm particle size, 10 × 100 mm; Waters, USA) was employed to purify the peptide-drug conjugate using acetonitrile:water as the mobile phase. A linear gradient was applied; 0 min, 10% acetonitrile; 30 min, 90% acetonitrile at 214 nm; the elution was collected and analysed.

### Cell growth inhibiting studies

The cytotoxicity of free paclitaxel and conjugate were determined by an MTT assay using HT-1080, Hep-2, Hep G2 and U-87MG cell lines. The cells (1 × 10^5^ cells/100 μl/well) were seeded in 96-well plates and cultured at 37°C and 5% CO_2_, respectively. Free paclitaxel or conjugate was incubated with cells at final concentrations of 0.1, 0.5, 1, 5, 10 and 100 μg/ml. The MTT solution (5 mg/ml in PBS) was titrated into each well after an incubation period of either 24, 48 or 72 hours. A traditional MTT protocol was performed, and cell viability was calculated.

### Scratch assay

The special function of MMP2 in cancer metastasis suggests that halt of MMPs activity is capable to cease the tumor cell metastasis. In this study, scratch assays were conducted in order to investigate whether the conjugate is able to inhibit the metastasis, given the increased paclitaxel concentration surrounding the tumour cells upon proteolysis by MMP2 specifically.

The HT-1080, U87MG, Hep G2 and Hep-2 cells were seeded in 6-well plates and cultured overnight. Following scraping of the cell layers in a straight line by a sterile p200 pipette tip, the debris was removed in two gentle rinses with PBS. Subsequently, 2.5 ml cell culture containing either free paclitaxel or conjuagte (final concentration at 5 μg/ml for paclitaxel) was plated. After 24 hours in a cell incubator, the distances between the two edges of the scratched cells were observed using Leica ICC50 HD microscopy (LEICA Microsystems Ltd, Switzerland). The apparent rate of inhibition was calculated using the following formula: inhibition rate (IR) = [(Distance_treated, 24 h_ - Distance_treated, 0 h_) - (Distance_control, 24 h_ - Distancecontrol, 0 h)]/Distance_treated, 0 h_ × 100.

### Drug release study upon MMP2 proteolysis

To determine whether the specific MMP2 proteolysis induced the release of paclitaxel or not, HPLC-MS was employed to monitor the conjugate dissociation at the presence of the MMP2. Free paclitaxel (100 μg) or conjugate (paclitaxel amount is 100 μg) solution was prepared in PBS buffer, pH 7.0, containing 100 μM ZnSO4 in various concentrations, following MMP2 (5 μg) titration. The release of free paclitaxel in the mixture at different time points (2 h, 4 h, 8 h, 12 h and 24 h) was monitored by RP-HPLC. A similar assay was also performed in cancer cell lines over-expressing MMP2 and in cancer cell lines without MMP2 expression.

### HPLC-MS measurement

Three ThermoFinnigan quadrupole mass spectrometers (TSQ7000) with XCALIBUR and LCQUAN software were equipped with an electrospray ion source and a divert valve. The chromatographic system consisted of an HPLC pump (model 616) and Controller 616 S from Waters (Milford, MA, USA) and an autosampler Series 200 from PerkinElmer (Norwalk, CT, USA). The Oasis HLB chromatography column (30 mm particle size, 1 × 50 mm) from Waters was used. Electrospray-ionisation was performed in positive ion mode. The heated capillary was set at 245°C and the spray voltage at 4.5 kV. Nitrogen was used as the sheath and auxiliary gas and set at 90 lb/square inch and 25 arbitrary units, respectively. The argon collision gas pressure was set to 2.5 mTorr. The samples (50 μl) were chromatographed at ambient temperature on the Oasis HLB column. Solvent A was acetonitrile, and Solvent B was 5 mM sodium trifluoroacetate in 0.1% aqueous formic acid. The analyses were chromatographed with a 0.8 ml/min flow rate with the following linear gradient: 0 min, 5% A; 0.5 min, 5% A; 5.5 min, 82% A. The gradient was followed by a 1.0 ml/min rinse for 1.0 min with 100% methanol and 30 s with 95% acetonitrile and by column re-equilibration to the initial conditions. The flow was diverted to the mass spectrometer for quantitative analysis.

### Treatment of human tumour xenograft mice

Six-week-old male BALB/c nude mice were obtained from Shanghai Laboratory Animal Co., China Academy of Sciences (Shanghai, China). Mice (weighing 16–20 mg) were housed in barrier facilities on a 12 h light/dark cycle. Food and water were supplied *ad libitum*. On day zero, two groups of mice were inoculated via i.p. injection with HT-1080 and U-87MG tumour cells (2 × 10^5^) in 0.5 ml of RPMI1640. Treatment was initiated after the tumour was allowed to grow to approximately 100 mm^3^ on the back of the mouse. This experimental protocol is intended to mimic the clinical situation when treatment begins after the tumour has already been established in the treatment of human tumour xenograft mice. The animals were treated with free paclitaxel or conjugate at the specified dose (15 mg/kg body weight) every 5 days. Control groups were treated with PVGLIG. During the experimental period of 30 days, the survival ratio was recorded in days after tumour injection. The mean and median survival time and the statistical significance of the results were determined by employing a two-tailed Wilcoxon's ranking test.

### Statistics

All data in this paper were presented as mean ± SD. The statistical significance was analysed using a two-tailed Wilcoxon's ranking test, and *P* < 0.05 was considered statistically significant.

## CONCLUSIONS

Paclitaxel is a widely used chemotherapeutic agent in the clinical treatment of various solid tumor including ovarian, breast, non-small cell lung, head and neck cancers. However, the off-target toxicity of paclitaxel restricted its clinical utility.

**Figure 5:**
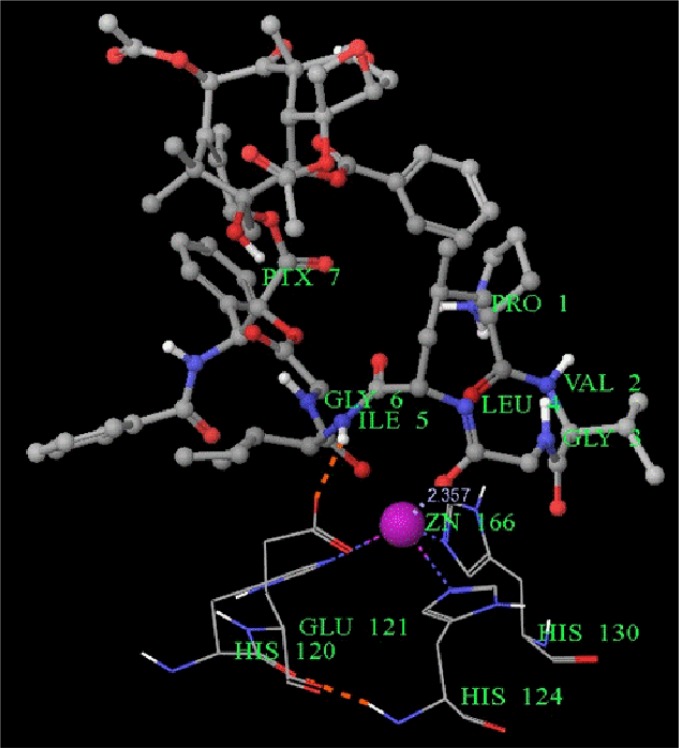
The molecular model of the interaction of conjugate and MMP2 protein Legend: The Leu4, Ile5 and Gly6 residues interact with Zn2+ binding site in MMP2 protein, the residue His120, His 124, His130 and His 166.

In this study, paclitaxel was successfully conjugated with a hexapeptide which is recognized by MMP2 specifically, the interaction of hexapeptide and MMP2 enzymatic pocket showed in Figure 5. Consequently, MMP2 enzymatically hydrolysed the hexapeptide and then release the paclitaxel molecule. This conjugate was considered to be a powerful approach for inhibiting the tumour growth and metastasis in tumor with high expression of MMP2.
